# Spatiotopic perceptual maps in humans: evidence from motion adaptation

**DOI:** 10.1098/rspb.2012.0637

**Published:** 2012-04-25

**Authors:** Marco Turi, David Burr

**Affiliations:** 1 Department of Physiological Sciences, Università Degli Studi di Pisa, Via S. Zeno 31, Pisa, Italy; 2 Department of Psychology, Università Degli Studi di Firenze, Firenze, Italy; 3 School of Psychology, University of Western Australia, Perth, Australia

**Keywords:** perception, spatial vision, visual stability

## Abstract

How our perceptual experience of the world remains stable and continuous despite the frequent repositioning eye movements remains very much a mystery. One possibility is that our brain actively constructs a *spatiotopic* representation of the world, which is anchored in external—or at least head-centred—coordinates. In this study, we show that the *positional motion aftereffect* (the change in apparent position after adaptation to motion) is spatially selective in external rather than retinal coordinates, whereas the classic motion aftereffect (the illusion of motion after prolonged inspection of a moving source) is selective in retinotopic coordinates. The results provide clear evidence for a spatiotopic map in humans: one which can be influenced by image motion.

## 1. Introduction

One of the greatest mysteries of visual neuroscience is how do we construct a stable representation of the external world from the sequence of retinal images produced as we scan our environment with eye, head and body movements. One possibility, suggested by many, is that there may exist in our brain a spatial representation encoded not in retinal, but in spatiotopic (or at least head-centred) coordinates, representing spatial location independently of where the eyes are looking. However, constructing maps of this sort poses a serious challenge to the visual system, necessitating the combination of retinal signals with eye-position signals [1]. Indeed, whether such neural maps actually exist remains a contentious issue.

### (a) Spatiotopicity

In a landmark study, Andersen and Mountcastle [2] showed that the excitability (or *gain fields*) of cells in the parietal cortex of macaque monkeys depend on gaze. This observation has been verified and demonstrated in much of visual cortex [3–5]. A series of studies has also shown that in many visual areas, including V6, ventral intraparietal cortex (VIP) and medial superior temporal (MST), neurons are truly *spatiotopic* [6–10]. However, in all cases only a small proportion of neurons showed spatiotopy, and not all studies have reported effects of this type.

Similarly, several functional imaging experiments have shown that gaze position can modulate BOLD responses in many human cortical areas [11–15]. Perhaps the clearest study was that of d'Avossa *et al.* [16], showing that gaze modulates the response of area middle temporal cortex + (MT+) (the presumed homologous region of monkey MT/MST in humans) in such a way as to create spatiotopic selectivity in screen coordinates, invariant for gaze shifts (while V1 is clearly retinotopically tuned). However, this result has been challenged by Gardner *et al.* [17], who claimed that BOLD responses in all of occipital cortex are tuned in retinotopic, not spatiotopic coordinates. Counter evidence has been provided by the Morrone group [18], showing that spatiotopy requires spatial attention, but the issue remains controversial.

Psychophysical evidence exists for spatiotopy, but this too is controversial. Melcher and Morrone [19] showed that motion signals integrated across eye movements in a spatiotopic (and also retinotopic) fashion. Melcher [20] has shown that several visual aftereffects—including spatial form and faces—have a spatiotopic component. Motion-induced adaptation to duration has been shown to be primarily spatiotopic when apparent motion is taken into account [21]; yet this too has been challenged ([22]; see also [23]). Recently, Zimmermann *et al*. [24] showed that under certain conditions ‘saccadic adaptation’ is spatially selective, in spatiotopic (external) coordinates. This not only points to the existence of a spatiotopic map that guides saccades, but also suggests that this map is constructed—or at least influenced by—eye movements.

### (b) Motion aftereffects

Encoding of spatial position can be influenced by many factors. For example, motion distorts space, displacing the apparent position of objects in the direction of motion [25,26]. Adaptation to motion also affects the perceived position: viewing a drifting grating or rotating windmill for some seconds causes subsequently viewed grating patches to appear to be displaced [27,28]. Given the evidence for spatiotopy in several motion areas [18], combined with the fact that motion influences spatial maps, one may expect motion adaptation to be spatiotopic. However, the famous *motion aftereffect* (MAE) [29,30] seems to be strictly retinotopic. Addams [29, p. 373] himself reported ‘Having steadfastly looked for a few seconds at a particular part of the cascade, admiring the confluence and decussation of the currents forming the liquid drapery of waters, and then suddenly directed my eyes to the left, to observe the vertical face of the sombre age-worn rocks immediately contiguous to the water-fall, I saw the rocky surface as if in motion upwards’. The illusory motion occurred after a leftward eye movement to the part of the retina that was previously stimulated by the motion of falls, transferring to the adjacent age-worn rocks: in other words, the effect was retinotopic, not spatiotopic. This observation, which has been confirmed by more formal techniques [21,31,32], seems to be at odds with the previously mentioned evidence for spatiotopic representation of motion.

However, it is unclear what neural levels generate the MAE. fMRI and electro-physiological evidence [33] suggest that it is present in MT, maybe earlier. Psychophysical studies also point to a low level of action. For example, the MAE does not transfer from luminance to chromatic stimuli, suggesting that it occurs before colour information is integrated [34].

The positional motion aftereffect (PMAE: the effect of adapting to motion on perceived position) seems to be distinct from the classic MAE. Whitney and Cavanagh [35] have demonstrated clear shifts in spatial position with no corresponding MAE. McKeefry *et al*. [34] have more convincing evidence: whereas the MAE is chromatically selective, motion-induced spatial distortions were completely insensitive to chromatic composition. The dissociation between chromatic selectivity of aftereffects suggested that chromatic inputs are segregated during initial analysis, but are later integrated, before the site where motion affects spatial position.

The PMAE, therefore, seems to occur at a higher level than the classic MAE, maybe a prime candidate for spatiotopy in motion-related representations of space. In this study, we investigated whether the PMAE may have a spatiotopic rather than a retinotopic coordinate base. The results confirm previous studies in showing that the classic MAE is strictly retinotopic, but show that the PMAE is spatiotopic, reinforcing the many previous studies implicating a spatiotopic analysis of visual motion.

## 2. Material and methods

### (a) Subjects

Seven observers participated in the experiment. All observers were naive of the objective of the experiment, except M.T. (an author). All had normal or corrected-to-normal vision.

### (b) Stimuli

The visual stimuli were presented in a dimly lit room on a 19-inch CRT monitor with 1024 × 768 resolution at a refresh rate of 100 Hz and mean luminance of 38 cd m^−2^. Subjects viewed the stimuli binocularly from a distance of 57 cm from the screen, with their chins resting on a chin-rest to reduce head movements. They were instructed to keep their heads directed towards the stimuli (verified by experimenter monitoring). Stimuli were generated and presented under Matlab v. 7.6 using PsychToolbox routines [36], linearized by careful gamma correction. Adapt and test stimuli were Gaussian-windowed sinusoidal gratings, modulated in luminance on a grey background (carrier frequency 1 cycles/degree, drift velocity 3° s^−1^: 3 Hz, Gaussian space constant 1°, contrast 0.9). By convention, rightward motion is considered positive and leftward negative.

Eye position was not monitored during the actual experiment, but each subject participated in a training session with similar stimulus sequences in a room equipped with eye-monitoring equipment (Eyelink 2000, SR Research, Canada) to verify their compliance and measure their saccades. During these sessions (about 20 trials per subject), all subjects saccaded normally. Latencies ranged from 170 to 220 ms (far less than the 500 ms pause between presentations). The primary saccade tended to undershoot (1° on average), but corrections brought the eyes within 30 arcmin of the saccade target within the 500 ms timeframe.

### (c) Traditional motion aftereffect

**Figure 1.**
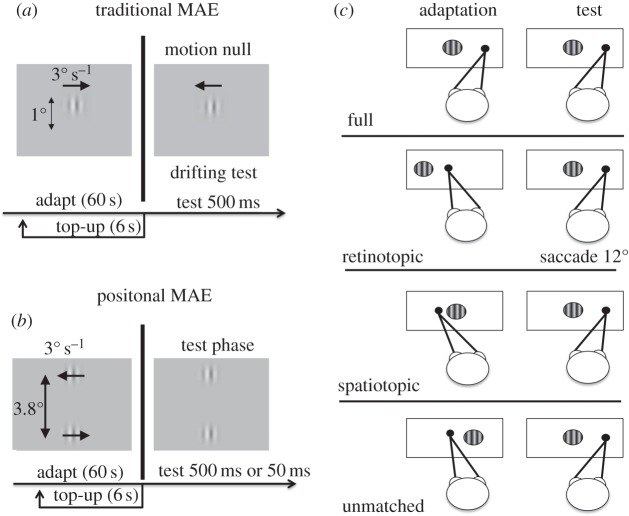
(*a*) Stimulus configuration for the traditional MAE. The subjects’ task was to indicate the direction of motion of the test. An adaptive algorithm adjusted the physical velocity of the test to home in on the null point. (*b*) Stimulus configuration for the PMAE. Subjects indicated which of the two test patches appeared more rightward. Again, an adaptive algorithm adjusted the physical positions of the stimuli to home in on the point of perceived alignment. (*c*) Experimental conditions. After adaptation four different conditions were tested: full adaptation, where no saccade occurred between the adaptation and the test, so both were in the same position in space and on the retina; spatiotopic, where the adaptor and test were in the same screen position but different retinal positions; retinotopic, in the same retinal but different screen positions; and unmatched, where the test was displayed at a location that was neither retinotopically nor spatiotopically adapted.

**Figure 2.**
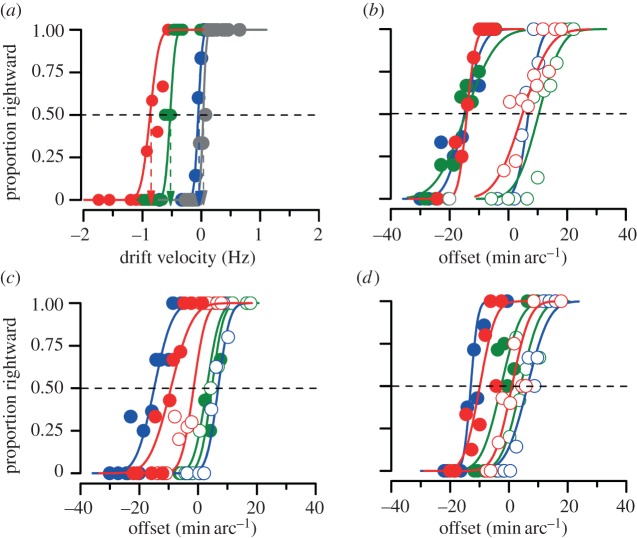
Example psychometric functions for one representative subject for the four different experiments. Conditions colour-coded (see legend). (*a*) Traditional MAE. (*b*) PMAE with a 500 ms stationary test stimulus. (*c*) PMAE with a 500 ms test stimulus drifting at a velocity that annuls the MAE. (*d*) PMAE with a 50 ms stationary test stimulus. Closed circles show the results for adaptation for leftward motion in the lower stimulus and open circles adaptation in the opposite direction (red, full; green, retinotopic; blue, spatiotopic and grey, unmatched).

The strength of the MAE was measured with a motion-nulling paradigm. At the start of each trial, observers viewed a rightward drifting adapting grating for 60 s, and again for a further 6 s before each trial (‘top up’). At 500 ms after extinction of the adaptor, a *test* grating patch was presented for 500 ms, in one of four possible conditions (figure 1). *Full-adaptation*: the test stimulus was presented at the same screen location as during adaptation, with no intervening saccade (so it was also the same location on the retina). *Retinotopic adaptation*: subjects made a 12° rightward saccade to a target displayed after extinction of adapting stimulus, and the test stimulus in the same retinotopic position (relative to fixation) as the adaptor. *Spatiotopic adaptation*: like retinotopic, except that the test grating was in the same screen position as the adaptor (hence different retinal positions). *Unmatched adaptation:* a 12° rightward saccade was made, with the test presented to a position that matched neither the screen nor retinotopic location of the adaptor (figure 1). The test gratings drifted at variable velocity, and the subject indicated whether they appeared to drift leftwards or rightwards (by key-press). The velocity of the test on each trial was varied by the adaptive Quest algorithm [37], which homed in on the point where the grating appeared to be stationary: point of subjective stationarity (PSS). To ensure a spread of speeds around the PSS, the QUEST estimate was jittered by adding to it a velocity drawn from a Gaussian distribution of standard deviation 0.5° s^−1^. The strength of the MAE was calculated by fitting the psychometric curves (like those of figure 2) with a best-fitting cumulative Gaussians functions, and calculating the mean to yield the estimated PSS (point of 50% rightward responses). As there was no bias in the PSS when tested without adaptation, this displacement from zero was taken as a measure of the effect magnitude.

### (d) Positional motion aftereffect

The *PMAE* was measured with two adaptation and test grating patches like those of the previous study, one centred 1.9° above the screen centre and the other 1.9° below. The patches drifted in opposite directions at 3° s^−1^, with the drift direction of lower patch pseudo-randomized between sessions. Otherwise the procedure was very similar to that used to measure the traditional aftereffect. There was again a 60 s period of initial adaptation and 6 s top-up periods before each trial. The test stimuli were presented for either 500 or 50 ms, in the same (horizontal) positions as those described above for the traditional MAE (figure 1). The test grating was either physically stationary (experiment 2), or drifted at a velocity to appear stationary (experiment 3: velocities set individually for each subject from the results of MAE). Subjects reported whether the upper grating appeared rightwards or leftwards of the lower grating.

The size of the positional shift was measured by symmetrically shifting the positions of the grating patches, one to the left and the other to the right. Again the amount of shift was determined on-line by the Quest algorithm (with 0.1° random jitter), homing in on the point where they appeared aligned. The point of subjective alignment was again calculated as the mean (50% point) of the fitted Gaussian curve. The strength of the position aftereffect was taken as the difference in PSA (point of subjective alignment) for the leftward and the rightward adaptation (upper patch). This minimized spatial-order effects (that were evident in informal testing without adaptation) and any other systematic biases. All the experimental conditions were tested in blocks, with the order of testing pseudo-randomized and counter-balanced between subjects. Thirty trials were run for each session, with two sessions per condition.

### (e) Statistical analysis: bootstrap sign tests

For the statistical comparison of experimental conditions we used two-tailed bootstrap sign tests [38], a technique that takes into account the error associated with each individual threshold as well as the between subject variance. A total of 10 000 iterations of bootstrap were run separately for each of the paired comparison. On each iteration, the data for each subject were independently sampled (with replacement), drawing 60 independent samples from the 60 data points, for that subject and that condition, and PSS or PSA calculated by fitting that sample with a cumulative Gaussian. The average PSSs or PSAs of all subjects for the two conditions were compared and scored. The *p* value was taken as the proportion of iterations where the condition A had higher PSS or PSA than condition B (condition B could be ‘zero’ when testing the significance of the effect).

The bootstrap test is powerful, as it considers both intra- and intersubject variability. However, we also performed standard *t*-test planned comparisons, which are broadly in agreement and tabled in the supplementary material.

## 3. Results

We first examined the spatial selectivity of the traditional MAE by motion-nulling task of a similar grating patch. Figure 2*a* shows sample psychometric functions of the annulling procedure for subject 1, under the four experimental conditions: full adaptation, where the adaptor and test coincided in both screen and retinal positions; spatiotopic alignment of the adaptor and test; retinotopic alignment; and unmatched, where the adaptor and test were neither spatiotopically nor retinotopically aligned (see electronic supplementary material  for all psychometric functions). The results are quite clear-cut. When the position adaptor and test were completely unmatched (in either coordinate system), the curve is centred on 0° s^−1^: leftward velocities seen leftward and rightward seen rightward. Adaptation to a grating drifting leftwards at –3° s^−1^ shifts the curve 0.8° s^−1^ to the left, yielding a PSS of –0.8° s^−1^: that is, the physical speed had to be –0.8° s^−1^ to appear stationary. This is the standard MAE. For our purposes, the interesting conditions are the spatiotopic and retinotopic paradigms. It is clear that the retinotopic condition produced a large MAE, while the spatiotopic condition produced none at all (consistent with Addams’ original observations). What mattered was the correspondence of retinal stimulation, not the position in space.

**Figure 3.**
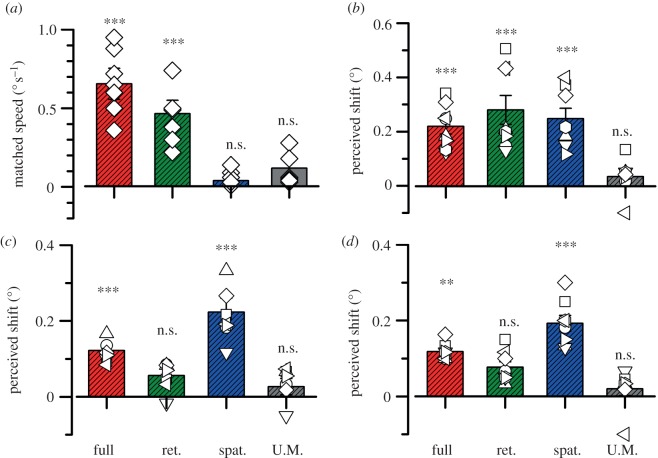
Average results for the four different experiments. Bars show group means and symbols show individual subjects. The conditions are colour-coded as before, from left to right: full, retinotopic, spatiotopic and unmatched. (*a*) Mean velocity needed to annul the traditional MAE. (*b*) PMAE defined for 500 ms stationary test stimulus. (*c*) PMAE with an apparently stationary 500 ms test stimulus (drifting at a velocity that annuls the MAE). (*d*) MAE for a 50 ms stationary test. The values of all conditions were tested for statistical difference from 0 by bootstrap sign test (see §2), with symbols above each bar showing the level of significance: n.s. *p* > 0.05; *p < 0.05; **p < 0.01 and ***p < 0.001).

From the psychometric functions of figure 2*a* (and those in electronic supplementary material), we calculated the point of perceived subjective stationarity (PSS), defined as the mean of the best-fitting Gaussian function. Figure 3*a* plots the PSSs for all subjects tested (symbols), and the group average (bars). Clearly, each subject showed strong MAEs in the full and retinotopic conditions, but weak or nil effects in the spatiotopic and unmatched conditions. For the statistical comparison of experimental conditions we used bootstrap sign tests (see §2), which showed that the full and retinotopic conditions were statistically significant from zero (*p* < 0.001, *p* < 0.01 respectively), while the spatiotopic and unadapted conditions were not (*p* = 0.40, *p* = 0.20), fully confirming previous results showing that the MAE is retinotopic [21,31,32].

However, the spatial selectivity of the positional MAE was quite different. Figure 2*b* shows psychometric functions for aligning the patches after adaptation to motion, under conditions similar to those in the previous experiment (500 ms stationary grating test patches). Closed circles refer to adaptation to rightward motion in the upper grating and leftward motion in the lower grating; open circles refer to motion in the opposite directions. The red data points show the full-adaptation condition, where there was both spatiotopic and retinotopic coincidence. Adaptation to leftward motion shifted the psychometric function leftwards (indicating that the perceived position of the patch was displaced rightwards), and adaptation to rightward motion shifted the curves in the other direction. The amount of shift was not exactly symmetrical, probably reflecting small field-dependent biases in spatial location localization. For this reason, we adapted separately in both directions of motion, and defined the magnitude of the effect as the difference in PSA for the two directions of motion (cancelling out any constant bias).

The green and blue symbols and curves show the results respectively for the retinotopic and spatiotopic conditions. Here the results differ from those for the classical MAE in that both conditions cause a positional aftereffect of similar magnitude. Figure 3*b* shows the magnitude of the positional MAE (defined as the difference in PSAs for the two motion conditions) for all subjects. All subjects showed the effect both in the spatiotopic and retinotopic conditions, and in all cases the effects are statistically significant (bootstrap sign tests: *p* < 10^−4^, *p* < 10^−3^, respectively). Neither the retinotopic nor the spatiotopic conditions were statistically different from the full-adaptation condition (*p* = 0.37, *p* = 0.39, respectively).

As the first experiment shows, adapting to motion causes a stationary grating to appear to drift in the opposite direction (the classical MAE). One possibility is that it is this apparent drift that causes the shift in position, much in the same way that real motion causes an apparent shift in position [25,26]. Indeed, Nishida & Johnston [27] have suggested that this could be the basis for the positional MAE. To test this possibility, we repeated the experiment using test stimuli where the MAE had been annulled, so the grating was perceived to be stationary (but physically moving), using the parameters obtained from the first experiment. Figure 2*c* shows psychometric functions for one observer, and figure 3*c* the group results. Under these conditions, with an apparently stationary test, the retinotopic effect disappeared, becoming statistically indistinguishable from zero (bootstrap sign test: *p* = 0.15). The magnitude of the effect with full adaptation was also reduced to about half the level with physically stationary stimuli compared with the 500 ms test condition (*p* = 0.002). However, the spatiotopic condition (where little motion cancellation was required) remained unchanged (*p* > 0.05). With speed-matched stimuli, the spatiotopic condition caused a significantly greater effect than did full adaptation (*p* = 0.02).

As a final test of the importance of apparent motion of the test stimulus, we used briefly presented (50 ms) stationary test gratings, too brief to convey a strong sense of apparent motion. The results of these test stimuli, shown in figures 2*d* and 3*d*, are similar to those of the speed-annulled stimuli: strong spatiotopic (*p* > 0.05) but weak retinotopic effects (*p* < 10^−4^), and reduced magnitude to the full-adaptation condition compared with the 500 ms test condition (*p* < 10^−4^). Again the spatiotopic condition caused a significantly greater effect than did full adaptation (*p* = 0.015).

## 4. Discussion

This study reports that adaptation to motion can be either retinotopic or spatiotopic. The traditional MAE—where stationary objects appear to drift in the opposite direction after adaptation to motion—is clearly retinotopic, agreeing with previous research [21,29,31,32]. However, the PMAE—where objects appear to be displaced after adaptation to motion—has a clear spatiotopic component. Indeed, when brief or apparently stationary stimuli are used to test it, the aftereffect seems to be entirely spatiotopic, with little or no retinotopic selectivity.

Previous evidence suggests that the two forms of aftereffect act at different neural levels of processing. The traditional MAE seems to modify fairly early levels of motion analysis, before chromatic and luminance signals are combined [34]. On the other hand, colour and luminance transfer completely with the PMAE, suggesting that this acts at a higher level of analysis, after colour and luminance motion signals are combined. Keefry *et al*. [34] suggested that it may act on MT, while the traditional MAE acts at an earlier level.

Our results show further that the two levels of analysis have different coordinate bases: the lower level is clearly eye-based, encoded in retinotopic coordinates that shift with each eye movement. However, the higher level of analysis tapped by the PMAE is spatiotopic (or at least craniotopic), encoded in screen-based (or at least head-based) coordinates. If Keefry *et al*.'s interpretation that they act respectively at areas V1 and MT is correct, it would be consistent with the imaging studies [16] showing clear retinotopy for the primary and the secondary visual cortex, and clear spatiotopy for areas MT and MST.

Our results clearly suggest two separate causes for the PMAE: one appears to be indirect, via the classical MAE, and the other directly adaptable in a spatiotopic frame. When the classical MAE is not cancelled—so the test grating appears to drift during the test phase—the positional MAE shows both retinotopic and spatiotopic effects. However, when the apparent motion is annulled, or minimized by using brief test stimuli, the retinotopic component is no longer measureable, suggesting that it arises indirectly from the apparent drift of the test grating. The magnitude of the ‘full’ adaptation condition (both retinotopic and spatiotopic) was significantly reduced (halved) with perceptually stationary tests. Nishida & Johnston [27] suggested that the PMAE resulted entirely from the apparent motion. However, our results suggest that this is only partially true: the classic MAE does indeed contribute to the positional aftereffect, but there is also a more direct effect, and this is spatiotopic. The pattern of results are similar to those observed with adaptation to time, where both a retinotopic and a spatiotopic effect are observed [21]. It is interesting that with apparently stationary or brief tests, the spatiotopic condition produced a significantly stronger effect than the full-adaptation condition. It is not clear why this should be so, but does suggest the existence of multiple maps: a retinotopic map that is not distorted by adaptation (only by apparent motion of the test stimuli) and a distortable spatiotopic map. In the full condition the two would be superimposed, and compete: the spatiotopic condition isolates the spatiotopic map.

The main conclusion to be drawn from results reported here—together with previous studies using adaptation techniques [21], saccade adaptation [24], subthreshold summation [19] and imaging [16,18]—is that there exists an explicit neural representation of space, in world-centred coordinates. It is not clear how this representation—or map—is constructed, but it is certain that it takes into account eye position information. That saccadic adaptation—change of saccade amplitude in response to false feedback—affects the spatiotopic map suggests that it may be built up from successive saccades. Certainly, the map shows a good deal of plasticity in that it can be readily adapted either by giving false feedback about saccadic landing [24] or, as this study shows, by prolonged exposure to motion. The functional role of the motion-induced distortion of space is far from clear, but is further evidence for clear interaction between motion and space.

## References

[1] Pouget A. Deneve S. Duhamel J. R. 2002 A computational perspective on the neural basis of multisensory spatial representations. Nat. Rev. Neurosci. 3, 741–747 10.1038/nrn914 (doi:10.1038/nrn914) 12209122

[2] Andersen R. A. Mountcastle V. B. 1983 The influence of the angle of gaze upon the excitability of the light-sensitive neurons of the posterior parietal cortex. J. Neurosci. 3, 532–548 6827308 10.1523/JNEUROSCI.03-03-00532.1983 PMC6564545

[3] Andersen R. A. Essick G. K. Siegel R. M. 1985 Encoding of spatial location by posterior parietal neurons. Science 230, 456–458 10.1126/science.4048942 (doi:10.1126/science.4048942) 4048942

[4] Bremmer F. Ilg U. J. Thiele A. Distler C. Hoffmann K. P. 1997 Eye position effects in monkey cortex. I. Visual and pursuit-related activity in extrastriate areas MT and MST. J. Neurophysiol. 77, 944–961 9065860 10.1152/jn.1997.77.2.944

[5] Bremmer F. Pouget A. Hoffmann K. P. 1998 Eye position encoding in the macaque posterior parietal cortex. Eur. J. Neurosci. 10, 153–160 10.1046/j.1460-9568.1998.00010.x (doi:10.1046/j.1460-9568.1998.00010.x) 9753122

[6] Bremmer F. Kubischik M. Pekel M. Hoffmann K. P. Lappe M. 2009 Visual selectivity for heading in monkey area MST. Exp. Brain Res. 200, 51–60 10.1007/s00221-009-1990-3 (doi:10.1007/s00221-009-1990-3) 19727690

[7] Duhamel J. R. Bremmer F. BenHamed S. Graf W. 1997 Spatial invariance of visual receptive fields in parietal cortex neurons. Nature 389, 845–848 10.1038/39865 (doi:10.1038/39865) 9349815

[8] Froehler M. T. Duffy C. J. 2002 Cortical neurons encoding path and place: where you go is where you are. Science 295, 2462–2465 10.1126/science.1067426 (doi:10.1126/science.1067426) 11923540

[9] Galletti C. Battaglini P. P. Fattori P. 1993 Parietal neurons encoding spatial locations in craniotopic coordinates. Exp. Brain Res. 96, 221–229 10.1016/S0079-6123(08)63269-0 (doi:10.1016/S0079-6123(08)63269-0) 8270019

[10] Ilg U. J. Schumann S. Thier P. 2004 Posterior parietal cortex neurons encode target motion in world-centered coordinates. Neuron 43, 145–151 10.1016/j.neuron.2004.06.006 (doi:10.1016/j.neuron.2004.06.006) 15233924

[11] DeSouza J. F. Dukelow S. P. Vilis T. 2002 Eye position signals modulate early dorsal and ventral visual areas. Cereb. Cortex 12, 991–997 10.1093/cercor/12.9.991 (doi:10.1093/cercor/12.9.991) 12183398

[12] Goossens J. Dukelow S. P. Menon R. S. Vilis T. van den Berg A. V. 2006 Representation of head-centric flow in the human motion complex. J. Neurosci. 26, 5616–5627 10.1523/JNEUROSCI.0730-06.2006 (doi:10.1523/JNEUROSCI.0730-06.2006) 16723518 PMC6675273

[13] McKyton A. Zohary E. 2007 Beyond retinotopic mapping: the spatial representation of objects in the human lateral occipital complex. Cereb. Cortex 17, 1164–1172 10.1093/cercor/bhl027 (doi:10.1093/cercor/bhl027) 16818474

[14] Merriam E. P. Genovese C. R. Colby C. L. 2003 Spatial updating in human parietal cortex. Neuron 39, 361–373 10.1016/S0896-6273(03)00393-3 (doi:10.1016/S0896-6273(03)00393-3) 12873391

[15] Sereno M. I. Huang R. S. 2006 A human parietal face area contains aligned head-centered visual and tactile maps. Nat. Neurosci. 9, 1337–1343 10.1038/nn1777 (doi:10.1038/nn1777) 16998482

[16] d'Avossa G. Tosetti M. Crespi S. Biagi L. Burr D. C. Morrone M. C. 2007 Spatiotopic selectivity of BOLD responses to visual motion in human area MT. Nat. Neurosci. 10, 249–255 10.1038/nn1824 (doi:10.1038/nn1824) 17195842

[17] Gardner J. L. Merriam E. P. Movshon J. A. Heeger D. J. 2008 Maps of visual space in human occipital cortex are retinotopic, not spatiotopic. J. Neurosci. 28, 3988–3999 10.1523/JNEUROSCI.5476-07.2008 (doi:10.1523/JNEUROSCI.5476-07.2008) 18400898 PMC2515359

[18] Crespi S. Biagi L. d'Avossa G. Burr D. C. Tosetti M. Morrone M. C. 2011 Spatiotopic coding of BOLD signal in human visual cortex depends on spatial attention. PLoS ONE 6, e21661 10.1371/journal.pone.0021661 (doi:10.1371/journal.pone.0021661) 21750720 PMC3131281

[19] Melcher D. Morrone M. C. 2003 Spatiotopic temporal integration of visual motion across saccadic eye movements. Nat. Neurosci. 6, 877–881 10.1038/nn1098 (doi:10.1038/nn1098) 12872128

[20] Melcher D. 2005 Spatiotopic transfer of visual-form adaptation across saccadic eye movements. Curr. Biol. 15, 1745–1748 10.1016/j.cub.2005.08.044 (doi:10.1016/j.cub.2005.08.044) 16213821

[21] Burr D. Tozzi A. Morrone M. C. 2007 Neural mechanisms for timing visual events are spatially selective in real-world coordinates. Nat. Neurosci. 10, 423–425 10.1038/nn1874 (doi:10.1038/nn1874) 17369824

[22] Bruno A. Ayhan I. Johnston A. 2011 Retinotopic adaptation-based visual duration compression. J. Vis. 10, 30 10.1167/10.10.30 (doi:10.1167/10.10.30) 20884495

[23] Burr D. C. Cicchini G. M. Arrighi R. Morrone M. C. 2011 Spatiotopic selectivity of adaptation-based compression of event duration. J. Vis. 11, 21 10.1167/11.2.21 (doi:10.1167/11.2.21) 21357369

[24] Zimmermann E. Burr D. Morrone M. C. 2011 Spatiotopic visual maps revealed by saccadic adaptation in humans. Curr. Biol. 21, 1380–1384 10.1016/j.cub.2011.06.014 (doi:10.1016/j.cub.2011.06.014) 21802296

[25] De Valois R. L. De Valois K. K. 1991 Vernier acuity with stationary moving Gabors. Vision Res. 31, 1619–1626 10.1016/0042-6989(91)90138-U (doi:10.1016/0042-6989(91)90138-U) 1949630

[26] Ramachandran V. S. Anstis S. M. 1990 Illusory displacement of equiluminous kinetic edges. Perception 19, 611–616 10.1068/p190611 (doi:10.1068/p190611) 2102995

[27] Nishida S. Johnston A. 1999 Influence of motion signals on the perceived position of spatial pattern. Nature 397, 610–612 10.1038/17600 (doi:10.1038/17600) 10050853

[28] Snowden R. J. 1998 Shifts in perceived position following adaptation to visual motion. Curr. Biol. 8, 1343–1345 10.1016/S0960-9822(07)00567-2 (doi:10.1016/S0960-9822(07)00567-2) 9843685

[29] Addams R. 1834 An account of a peculiar optical phaenomenon after having looked at a moving body. Lond. Edinburgh Philosoph. Mag. J. Sci. 5, 373–374

[30] Wohlgemuth A. 1911 On the after-effect of seen movement. Br. J. Psychol. 1 (Suppl.), 1–117

[31] Knapen T. Rolfs M. Cavanagh P. 2009 The reference frame of the motion aftereffect is retinotopic. J. Vis. 9, 11–17 10.1167/9.5.16 (doi:10.1167/9.5.16). 19757894

[32] Wenderoth P. Wiese M. 2008 Retinotopic encoding of the direction aftereffect. Vis. Res. 48, 1949–1954 10.1016/j.visres.2008.06.013 (doi:10.1016/j.visres.2008.06.013) 18621074

[33] Tootell R. B. Reppas J. B. Dale A. M. Look R. B. Sereno M. I. Malach R. Brady T. J. Rosen B. R. 1995 Visual motion aftereffect in human cortical area MT revealed by functional magnetic resonance imaging. Nature 375, 139–141 10.1038/375139a0 (doi:10.1038/375139a0) 7753168

[34] McKeefry D. J. Laviers E. G. McGraw P. V. 2006 The segregation and integration of colour in motion processing revealed by motion after-effects. Proc. R. Soc. B 273, 91–99 10.1098/rspb.2005.3293 (doi:10.1098/rspb.2005.3293) PMC1560013 16519240

[35] Whitney D. Cavanagh P. 2003 Motion adaptation shifts apparent position without the motion aftereffect. Percept. Psychophys. 65, 1011–1018 10.3758/BF03194830 (doi:10.3758/BF03194830) 14674629

[36] Brainard D. H. 1997 The psychophysics toolbox. Spat. Vis. 10, 433–436 10.1163/156856897X00357 (doi:10.1163/156856897X00357) 9176952

[37] Watson A. B. Pelli D. G. 1983 QUEST: a Bayesian adaptive psychometric method. Percept. Psychophys. 33, 113–120 10.3758/BF03202828 (doi:10.3758/BF03202828) 6844102

[38] Efron B. Tibshirani R. J. 1993 An introduction to the bootstrap. New York, London: Chapman & Hall

